# 

**Published:** 1908-10-15

**Authors:** 


CONTENTS

Event and Comment - - - - - - - - 487
Salient Points in the Histology of the Tooth, *By Russell Bunting, D.D.S.* 489
Interstitial Gingivitis, - *By Edward Cornelius Briggs, M. D.* 499
Recent Studies on Novocain, *By Richard H. Riethmuller, Ph.D.* 515
Anesthesia by Intra-Alveolar Injection, *By F. L. Fossume, D.D.S.* 528
What Causes the Dentin to be Sensitive, *By C. F. Kabell, D.D.S.* 530
Bibliology - - - - - - - - - - - - 533
Indents - - - - - - - - - - - - - 537
Announcements - - - - - - - - - - 533

